# Snowball Vs. House-to-House Technique for Measuring Annual Incidence of Kala-azar in the Higher Endemic Blocks of Bihar, India: A Comparison

**DOI:** 10.1371/journal.pntd.0004970

**Published:** 2016-09-28

**Authors:** Niyamat A. Siddiqui, Vidya N. Rabidas, Sanjay K. Sinha, Rakesh B. Verma, Krishna Pandey, Vijay P. Singh, Alok Ranjan, Roshan K. Topno, Chandra S. Lal, Vijay Kumar, Ganesh C. Sahoo, Srikantaih Sridhar, Arvind Pandey, Pradeep Das

**Affiliations:** 1 Rajendra Memorial Research Institute of Medical Sciences (ICMR), Agamkuan, Patna, Bihar, India; 2 All India Institute of Medical Sciences (AIIMS), Patna, Bihar, India; 3 CARE India, Bihar, India; 4 National Institute of Medical Statistics (ICMR), Ansari Nagar, New Delhi India; Institut Pasteur de Tunis, TUNISIA

## Abstract

**Background:**

Visceral Leishmaniasis, commonly known as kala-azar, is widely prevalent in Bihar. The National Kala-azar Control Program has applied house-to-house survey approach several times for estimating Kala-azar incidence in the past. However, this approach includes huge logistics and operational cost, as occurrence of kala-azar is clustered in nature. The present study aims to compare efficiency, cost and feasibility of snowball sampling approach to house-to-house survey approach in capturing kala-azar cases in two endemic districts of Bihar, India.

**Methodology/Principal findings:**

A community based cross-sectional study was conducted in two highly endemic Primary Health Centre (PHC) areas, each from two endemic districts of Bihar, India. Snowball technique (used to locate potential subjects with help of key informants where subjects are hard to locate) and house-to-house survey technique were applied to detect all the new cases of Kala-azar during a defined reference period of one year i.e. June, 2010 to May, 2011. The study covered a total of 105,035 households with 537,153 populations. Out of total 561 cases and 17 deaths probably due to kala-azar, identified by the study, snowball sampling approach captured only 221 cases and 13 deaths, whereas 489 cases and 17 deaths were detected by house-to-house survey approach. Higher value of McNemar’s χ² statistics (64; p<0.0001) for house-to-house survey approach than snowball sampling and relative difference (>1) indicates that most of the kala-azar cases missed by snowball sampling were captured by house-to-house approach with 13% of omission.

**Conclusion/Significance:**

Snowball sampling was not found sensitive enough as it captured only about 50% of VL cases. However, it captured about 77% of the deaths probably due to kala-azar and was found more cost-effective than house-to-house approach. Standardization of snowball approach with improved procedure, training and logistics may enhance the sensitivity of snowball sampling and its application in national Kala-azar elimination programme as cost-effective approach for estimation of kala-azar burden.

## Author Summary

Visceral Leishmaniasis, commonly known as kala-azar, is one of the major health concerns for Bihar state in India. Time to time estimation of kala-azar incidence plays a pivotal role in defining and evaluating control strategies under National Kala-azar Elimination programme. The house-to-house survey approach, adopted several times for estimation of kala-azar incidence, requires huge logistics and operational cost as occurrence of kala-azar is clustered in nature. Snowball sampling approach is used to locate potential subjects with help of key informants where subjects are hard to locate. This is the first ever comparative study to assess efficacy, cost and feasibility of snowball sampling approach to house-to-house survey in capturing kala-azar cases. Snowball sampling was found cost-effective, but not sensitive enough as it captured about 50% of kala-azar cases. It captured deaths possibly due to kala-azar more efficiently (77%). However, standardization of snowball approach with improved procedure, training, logistics, etc. may enhance the sensitivity of snowball sampling and its application in national Kala-azar elimination programme as cost-effective approach for estimation of kala-azar burden.

## Introduction

Visceral Leishmaniasis (VL), also known as Kala-azar, is a major cause of morbidity and mortality and of tremendous public health importance in India, Bangladesh and Nepal affecting the poorest population groups, primarily in rural areas. With an estimated 200 million people at risk, India, Nepal and Bangladesh harbour an estimated 67% of the global VL disease burden [1]. In India, Bihar state alone reports nearly 80–90% of the VL cases. In this region, *Leishmania donovani* is the only species causing VL, the female sand fly *Phlebotomus argentipes* is the only vector and humans are the only known reservoir [2–3]. Recent advances in rapid field based diagnostics and availability of effective and safe oral drugs make the disease a target for elimination (reducing incidence to less than one per 10,000 population at sub-district level by 2020) [4]. The country is under kala-azar elimination mode, therefore reduction in kala-azar incidence has been the key developmental goal for monitoring the progress of the national elimination program. The need of setting such goals has been discussed and documented in various national and international deliberations and brain storming meetings at Office of the Directorate, National Vector Borne Disease Control Program, New Delhi (NVBDCP) [5, 6]. Availability of reliable data is one of the major concerns. It is said that measuring VL incidence is not only crucial but also complex for practical reasons. NVBDCP provides state-wise reported number of kala-azar cases and deaths in India [7]. But the estimate of kala-azar disease burden is suspected to be under-estimated in absence of a robust surveillance mechanism [8]. Since this disease is rare with clustered distribution, its estimation with adequate precision requires a very large sample for survey that is logistically and financially burdensome.

As the kala-azar control program moves to an elimination trajectory, the program has set itself the goal of reducing the annual incidence of kala-azar to less than 1 per 10,000 populations in at least half the currently affected blocks by the year 2017. The estimate of VL incidence with high precision is a major issue for impact assessment of elimination strategies. The current “gold standard” method for estimating disease burden is house-to-house approach (HHA) that requires high logistic and operational cost. Snowball sampling approach (SBA) could be an alternative to HHA. Snowball sampling is preferred when the desired sample characteristic is rare, difficult and/or cost restrictive to locate potential subjects in the study population. Initially, key informants for the study population are identified, and based on the information provided by the key informants, potential subjects are located. The identified potential subjects may provide information about other subject/s in turn and so on. SBA relies on referrals from initial subjects to generate additional subjects. This approach has widely been used to identify criminals, prostitutes [9], drug users [10–12] and people with unusual or stigmatized conditions such as HIV/AIDS, STD patients in a defined study population [13]. Given the inaccessibility of these populations, the referrals can significantly facilitate researchers to locate potential subjects through their social networks. However, this approach is biased and may reduce the likelihood of sample representing good cross-section from the population. But on the other side, it can enormously lower search costs. Nonetheless the method has been found to be economical, efficient and effective as compared to other probabilistic approaches of sampling applied in course of investigating rare events [14, 15]. In India, it has been used for estimating maternal-mortality ratio in the population [16]. In theory, this is an efficient and cost-effective method to find cases of rare diseases. It is most useful where the condition or event under consideration is well-recognized by communities. Kala-azar is familiar to communities in Bihar, at least in the endemic areas, and thus it appeared that SBA should work. This is the first ever study to assess whether SBA can be applied for measuring annual incidence of kala-azar.

## Materials and Methods

Based on recommendation of a high level consultative meeting on “Indicators for kala-azar elimination”, held at the Office of WHO/SEARO, New Delhi in June 2009, this community-based cross-sectional study was undertaken on pilot basis to compare efficacy, cost and feasibility of snowball sampling approach with house-to-house approach for measuring kala-azar incidence in a pre-defined highly endemic population of Bihar, India.

### Study setting, design and sampling

The study was conducted in two socio-culturally distinct highly endemic districts, namely Vaishali and Saharsa, of Bihar, India. Vaishali is situated in northern region of Bihar whereas Saharsa is one of the north-eastern districts, close to the border of West Bengal, other kala-azar endemic state. Apart from endemic status, other criteria considered for district selection were: a) Cultural distinctiveness (which could provide assurance of the method being effective in varying cultural contexts), b) Variation in health care access (which could determine the level of awareness of the disease, and influence the usefulness of key informants within the health system), and c) Inclusion of distinct logistical contexts (which could be helpful in preparation of larg baseline survey).

From each of the two districts, one of the most highly endemic Public Health Centre (PHC) was selected. Lalganj PHC of Vaishali district had relatively good, long-established health care access and health facilities than other selected PHC (Simri-Bakhtiyarpur) of Sahrasa district. All the villages, with varying level of endemic status, under the selected PHC constitute the study area.

A community based cross-sectional study was designed to cover overall population of the two PHCs. Both house-to-house and snowball sampling approach were applied by two separate teams to capture cases of kala-azar during a defined reference period of one year. Snowball sampling preceded the house-to-house survey. All the suspected cases of kala-azar, identified through both approaches, were enlisted for further confirmation. Details of cost and time involved in data collection by both approaches were also documented for cost-efficacy analysis.

A multistage sampling technique was adopted. In the second stage, the most highly endemic PHC from each selected district was selected, and at the third stage villages and households under PHC areas were enumerated.

The relatively low incidence of kala-azar and its clustering nature implied that required sample sizes could be very large, close to the actual population of the PHC/block. Every habitation in the PHC was covered by both approaches.

The study was for a period of 18 months. However, the information on kala-azar cases occurred in between June 2010 to May 2011 was captured under this study.

### Survey procedure

Prior to actual survey, mapping and listing of all the households under study area were performed by trained staffs. It was started in 2^nd^ week of January 2011 and completed by 2^nd^ week of March 2011.

### Case definitions

A kala-azar case may be defined as: An individual with fever of more than 15 days duration with splenomegaly and positive by rapid diagnostic blood test (rk-39 strip test), or a case confirmed with laboratory-based diagnosis i.e. splenic/ bone marrow aspirate examination for *Leishmania donovani* (L.d.). In context of measuring annual incidence, the following criteria were considered during survey (implied for both approach) to be a case of kala-azar:

- A person with history of fever within the reference period and diagnosed/ treated for kala-azar (subject to document verification during the survey).
- A person with history of prolonged fever within the reference period, but did not have any document for diagnosis/ treatment (subject to positive by rk-39 strip test conducted during the survey).
- A current case of kala-azar (subject to document verification during the survey)
- A current case of fever, not tested as on date (subject to positive by rk-39 strip test during the survey)

Death probably due to kala-azar was considered if the individual died during or before treatment with history of prolonged fever.

During survey, a popular festival in Bihar (known as “Chhat”) was used to identify the reference period of “the last one year” (defined as last Chhat to current Chhat) to ensure minimal misclassification of incidence across consecutive years. To ensure non-missing of deaths probably due to kala-azar, each and every households having death during the reference period were visited. The head/ representative of household was interviewed to collect detail information on death/s occurred, using the standardized verbal autopsy schedule used in earlier study conducted by Padam Singh *et*. *al*., 2007. Though the schedule was administered by a trained field supervisor, relation of death with kala-azar was verified through respective medical officer and renowned local person, wherever it was possible, and finally judged by study physician.

### Use of survey instruments and data collection

House-to-house and snowballing sampling approach was made by separate teams of trained field workers. It was assumed that an intensive house-to-house survey for kala-azar may increase people’s awareness, and eventually may positively affect the sensitivity of snowballing approach in identification of cases and deaths. In order to overrun this bias, snowballing survey was preceded to house-to-house survey.

### Snowball sampling approach

Area-wise list of Accredited Social Health Activist (ASHA’s), Aganwadi Workers (AWW’s), Auxiliary Nurse Midwife (ANM’s), school teachers, local healers and local leaders were prepared as possible key informants, but it was not limited to them only. A pilot field visit suggested that mere these key informants may not suffice to track all kala-azar cases, and few key informants may not be available at the time of actual survey by the snowballing team. Typically, communities live in caste-based clusters (locally called tolas), and their inter-cluster social interactions are not intensive enough to provide information on kala-azar from other cluster. Hence, additional key informants were also identified through a series of focussed group discussion meetings conducted in different areas (S1 File).

The snowballing team contacted the identified key informants for the area to be surveyed. Based on their information, the kala-azar affected households were visited. A detailed interview schedule, specifically designed to capture the case/ death as per study definition, was administered to the head/ representative of households. Apart from case and death, data relevant to the family’s basic socio-demographic background was also collected. Besides the key informants, information provided by members of the visited households also provided a link to other households possibly having kala-azar during the reference period. On an average, three villages were covered by one team per day as 2–3 hours were required to cover one average sized village.

### House-to-house survey approach

Each and every households of the study area were covered for house-to house survey to identify kala-azar cases occurred within the reference period. In order to ensure thorough coverage of all the households, standard principle of house-listing operations was followed. Using right-hand/ left-hand rule, each households were marked with unique household numbers and a rough map was drawn for household tracking. Head of the households or adult representative (in case of absence of head) were interviewed through pre-tested interview schedule to collect demographic data and information on individual(s) who suffered from/ died possibly due to kala-azar during the reference period. Similar to snowballing sampling approach, a detailed interview schedule was used to confirm the case as per study definition, and capture socio-demographic data of the family. A team of four investigators and one supervisor completely covered one village per working day.

Both, snowballing and house-to-house survey teams were provided sub-centre wise list of villages for each PHC. In order to ensure thorough coverage, teams were instructed to cover all villages of the assigned sub-centre prior to moving to villages of other sub-centres. Special attention was made to ensure coverage of all the outlying clusters/ hamlets of villages.

### Final list of suspected cases and deaths identified

The central team of investigators closely monitored the thorough coverage of study population and data collection through both approaches (SBA and HHA), with special emphasis on identified potential suspected cases and deaths. After cross verification, village-wise final list of suspected cases was generated for confirmation by the medical field team. A separate list of deaths possibly due to kala-azar during the reference period, identified by both approaches, was also prepared. Each and every identified cases and deaths were marked with the type of approach through which identified i.e. HHA, SBA or both for further analysis.

### Confirmation of identified cases and deaths

Village-wise identified cases and deaths were confirmed by medical team consisted of one clinician, one technician and one paramedics of Rajendra Memorial Research Institute of Medical Sciences (RMRIMS). During door-level visit, the team re-verified the fact through personnel discussion and the available medical documents. After taking informed consent from the concerned individuals/ legal representatives, the enlisted cases were clinically examined and subjected to on-the-spot rapid diagnostic blood test (rk-39 strip test). Since rK-39 test is sensitive enough to capture present as well as past infection of leishmania, rK-39 positive cases were considered as true cases identified during survey. Some rK-39 negative cases were considered based on strong document-based evidence. The cause of death was judged by study physician using verbal autopsy schedule supported by documentary evidence (if available).

### Logistics

#### Field teams

Separate field teams for snowballing and house-to-house approach were deployed to avoid inter-mixing of data and personal influence. For house-to-house survey, 80 field investigators and 20 supervisors were hired. Minimum education qualification for field investigators and supervisors was matriculate (Class 10 pass) and graduate degree (preferably in science subject) respectively with experience in field based activity. Each team consisted of four investigators and one supervisor and two teams moved together by a hired four-wheel vehicle to cover the target population of different villages in same direction.

Field investigators hired for snowballing survey were graduate with experience in field work. Investigators having their own two-wheel vehicle (motorcycle) were preferred. Apart from field investigators, 20 senior level investigators were also hired. Field work of snowballing team was monitored closely by independent observers nominated for the study. A team of two senior level investigators moved together in the field for highest mobility and cost effectiveness. They were compensated for POL and any breakdowns occurred during field visit.

Proportionate number of teams was constituted for snowballing and house-to-house approach to maintain more or less uniform time lag (about a week) between the two teams visiting a given village. Field investigators were hired locally at PHC/ block level whereas supervisors and senior level investigators were hired at district level.

#### Training

A brief one-day class-room training at RMRIMS, followed by 3-days hands-on training in field, was conducted for field investigators and supervisors, hired for house-to-house survey, to make them fully comfortable with the tools and working procedures. Field-based hands-on training was arranged in kala-azar endemic villages other than the study area and it was focussed on skill development in identification of suspected cases/ deaths. Training was imparted by research team in three batches (maximum 40 trainees per batch including about 30 field investigators and 8 supervisors). Supervisors were specifically trained on quality control measures to make them competent enough for data verification related to suspected cases/ deaths.

Being snowball sampling a new approach for kala-azar survey, personnel deployed for this approach were imparted more intensive training than the house-to-house team. A thorough classroom session, conducted at RMRIMS, on kala-azar orientation and study-specific methodologies was followed by 6-days field-based hands-on training in different kala-azar endemic villages. The add-on training component for senior level investigators was supervision and monitoring skill development.

The personnel hired at different levels for both the approaches were deployed in actual survey only when they were certified by trainers as competent enough to accomplish their assigned task. A detailed record of training activities including expenditure incurred was maintained to measure realistic estimates of efforts required for such kind of survey in future if planned by the Government.

### Data management, quality control and statistical analysis

The total data load was small compared to other surveys of equivalent size, since the instruments were limited in size. After field editing (by field supervisors) and desk editing (by designated desk editors), data was entered in customized data entry programs in Epi Info Version 3.3.2 (CDC Atlanta). All forms of suspected cases were double-entered and errors validated against original schedules. The snowballing team was not having any field supervisors accompanying them, and thus checked and balanced from outside. Part of this was provided by the supervisors of the house-to-house team that followed the snowballing team. They were confirmed whether snowballing was attempted in the village. In addition, two field teams from RMRI were looked into the entire data collection process using independent vehicles, and were conducted systematic back checks on both, snowballing and house-to-house teams, making particular efforts to find additional cases in a sub-sample of villages. Finally, the medical teams that visit suspected cases was reconfirmed that they do indeed meet the definition of suspected cases. All the structured/semi-structured questionnaires used under this survey were pre-tested.

Statistical analysis was done using Epi Info Version 3.3.2 (CDC Atlanta). For comparison of snowball technique with house-to-house survey, the number of kala-azar cases identified in the study area through each approach was matched. We conducted matched-pair analysis (using McNemar χ² statistic) to find a statistical significance as well as identify number of cases/ deaths missed by house-to-house approach as compared to the snowball approach and vice versa. The population of affected villages in sub-district/PHC level was used as denominator for estimation of incidence. Per unit cost and time involved in snowballing approach was compared with that of house-to-house approach to assess its cost-effectiveness. Descriptive statistics as well as chi square test was used for comparison between proportions. In addition to that, Standard Error (SE), Relative precision (RP) and 95% Confidence Interval (CI) were calculated for assessing the estimates.

### Ethics statement

The study was duly approved by the Scientific Advisory Committee and Ethics Committee of Rajendra Memorial Research Institute of Medical Sciences. A written informed consent was obtained from all adult subjects participating in the study. In case of study subjects less than 18 years of age, written consent was obtained from their parent or guardian. Confidentiality was maintained as per the ethical norm.

## Results

### Brief profile of the study districts

Vaishali consists of three sub-divisions and 16 blocks/PHCs with Hajipur Sadar as district head quarter. Vaishali is an important pilgrim center for both Buddhists and Jains and surrounded by banana and mango groves as well as rice fields. It is 30 kilometers from state capital and just after crossing river Ganga. Vaishali stretches in 2036 sq km area with latitude = 25.11’ N and longitude = 85.32’ E. The total population of district is 3,495,249 with 1,847,049 males and 1,648,191 females, literacy rate as 66.60 and sex ratio of 895/1000. The population density is 1,717 inhabitants per square kilometer and the population growth rate over the decade 2001–2011 was 28.57%.

**Fig 1:**
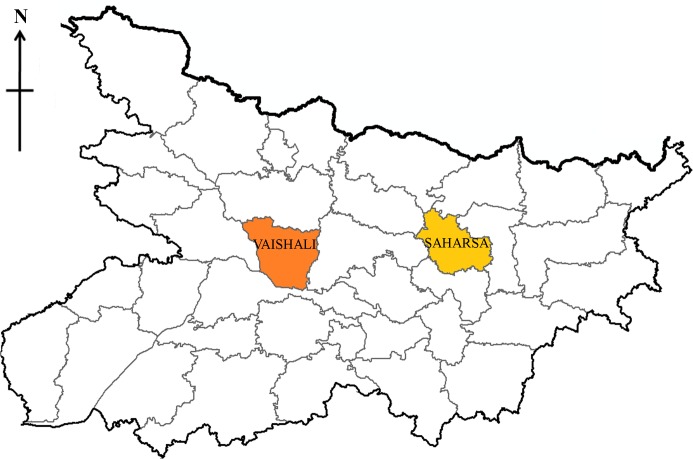
Map of Bihar showing study districts (Source: www.bihar.gov.in).

There are two sub-divisions and 10 blocks/PHCs in Saharsa district with Saharsa Sadar and Simri-Bakhtiyarpur as district head quarter. Saharsa district is surrounded on the west by the river Koshi. Saharsa is located 300 Km away from state capital Patna and stretching in 1696 sq km area with latitude = 25.88’ N and longitude = 86.60’ E. As per census 2011, Saharsa has a population of 1,900,661 with 997,174 males and 903,487 females, literacy rate as 54.57 and sex ratio of 906/1000. The population density is 1,125 inhabitants per square kilometre and the population growth rate over the decade 2001–2011 was 25.79% (Fig 1).

### Findings

Two approaches (snowballing and house-to-house survey) were used to collect data on morbidity and mortality of kala-azar in the catchment area of two PHCs, one from each of the two socio-cultural distinct districts. A total of 105,035 households with 537,153 populations [(Male 285,617 (53%) and female 251,536 (47%)] spread over 213 villages under area of 58 sub-centres were covered. The socio-demographic profile and age-sex distribution of the screened population was consistent with that of census 2001 and 2011 of the study districts [17]. The average household size of 5 was comparable to that of the national average of Bihar state.

During the reference period, a total of 489 and 221 confirmed cases of kala-azar were detected by HHA and SBA respectively (Table 1). Matched-pair analysis using McNemar’s χ² statistics revealed that out of 221 cases identified by SBA, 149 matched with cases identified by HHA and rest 72 cases were missed by HHA. Considering additional cases captured by SBA (N = 72), the total number of confirmed kala-azar in the study population came to 561. Thus efficacy of HHA and SBA in capturing kala-azar cases was 87% (489 of 561) and 39% (221 of 561) respectively. Though, HHA is supposed to be gold standard, it also missed about 13% of true cases. Out of 17 deaths possibly due to kala-azar, captured by HHA, 13 (76%) were captured by SBA. No additional deaths were detected by SBA. Snowballing detected deaths more efficiently (13 of 17, 76%) than cases (221 of 561, 39%). The higher value of McNemar’s χ² statistics (64; p<0.0001) with relative difference >1 indicated that most of the kala-azar cases missed by snowballing approach were captured by house-to-house approach with some percentage of omission. Snowball sampling approach was not found sensitive enough for measuring annual incidence (reference period of one year) of kala-azar as compared to house-to-house approach. House-to-house approach picked up about 48% more cases than snowballing approach. However, snowballing approach was able to accurately capture about 50% of true cases.

**Table 1:** Number of Kala-azar cases captured by snowballing and house-to-house survey approach.

PHC (District)	Number of Kala-azar cases	
	Snowballing (n)	House-to-house (n)
Lalganj (Vaishali)	43	92
Simri Bakhtiyarpur (Saharsa)	178	397
Total	221	489

The house-to-house approach was completed in 37 days with 40 field staff resulting into 1480 man-days per PHC whereas in snowballing approach, only 10 field staff covered the whole study population in 24 days *i*.*e*. 240 man-days per PHC. The ratio was found as 1:6. As regard to cost, INR 737,000 was incurred on house-to-house approach per PHC while cost for snowballing approach per PHC came to INR 370,300, about 50% less than house-to-house approach. Considering accurately capturing of about 50% of cases and 76% of deaths, SBA may be considered as cost-effective alternative to house-to-house approach.

Based on total number of kala-azar cases cumulatively identified by both approaches under this study, the estimated incidence in Lalganj (Vaishali district) and Simri Bakhtiyarpur PHC (Saharsa district) was 3.6 and 16.6 per 10,000 respectively. This estimate was compared with officially reported surveillance data of the respective PHCs during the reference period. The estimated incidence was found almost similar to officially reported incidence in Lalganj PHC (3.6 vs. 3.2), but it was significantly higher in Simari Bakhtiyarpur (16.6 vs. 7.1) (p<0.05). Although the block sample covered in the survey was below the size required for estimating the annual kala-azar incidence in the higher endemic blocks of Bihar. But it was further tried to provide the possible estimates of annual incidence of kala-azar based on the findings of this study along with its standard error and confidence interval for the selected blocks and as well for all the higher endemic blocks of state (Table 2).

**Table 2:** Estimates of annual incidence of Kala-azar in the study area based on total number of cases captured during survey.

PHC (District)	Population covered (N)	Kala-azar cases captured (N)	Incidence /10 000 population	SE (Incidence)	Relative Precision (Incidence)	95% C.I.	
						Lower	Upper
Lalganj (Vaishali)	254597	92	3.6	0.61	34.78	2.25	4.64
Simri Bakhtiyarpur (Saharsa)	282556	469	16.6	11.81	60.29	15.24	61.55
Total	537153	561	10.4	5.86	67.99	5.42	28.38

SE: Standard error; C.I.: Confidence interval

Under snowballing approach, altogether 1501 key informants were identified, of which maximum key informants 913 (61%) were from others categories that includes shopkeepers, big agricultural farmers (renowned home owner), pharmacist, fellow villagers, relatives, etc. A statistically significant difference (p<0.001) was observed between other categories of key informants and defined (accredited) categories of key informants (viz. AWW, ASHA, Sarpanch, local healer, teacher, etc.) in respect to giving information about kala-azar cases and deaths. Amongst the defined categories of key informants, ASHAs and school teachers performed poor in providing reference to cases/deaths (1.85–7.02% and 0.67–3.70% respectively). However, comparatively ASHAs were found more effective (7.02%) than school teacher (0%) in identifying deaths during the reference period of last one year. The role of the village leaders/Sarpanch in identification of kala-azar cases as key informant was found negligible (1.20%).

**Fig 2:**
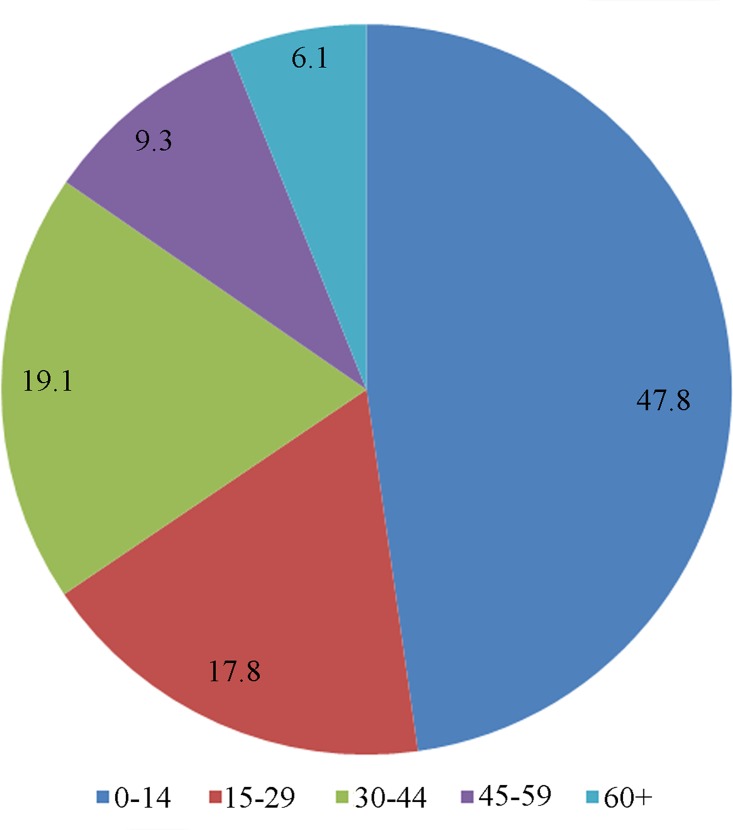
Age group distribution of kala-azar cases identified during survey.

Socio-demographic characteristics of kala-azar cases identified during the survey revealed that significantly higher proportion (268, 48%) of cases were from 0–14 years of age as compared to rest age-groups viz. 15–29, 30–44 & 45–59 years (p<0.001) (Fig 2). About half of the cases were from growing age group and majority (333, 59%) were males. kala-azar patients coming from very low socio-economic categories (viz. unskilled labourers, rickshaw pullers, Agricultural labourer etc.) were significantly higher (78%) than altogether middle and higher socio-economic categories (p<0.001).

The concordance between Health Management Information System (HMIS) and house-to-house data revealed that about 75% of cases detected by house-to-house approach were completely and accurately matched with HMIS data and rest 25% cases were not reported to HMIS.

## Discussion

Under the national kala-azar elimination programme, Health Management Information System (HMIS) provides the information on kala-azar cases and deaths at different levels viz. PHC, district, state and national level. The estimated incidence of kala-azar, inferred from this study, was 3.6 and 16.6 per 10,000 populations for Lalganj (Vaishali district) and Simri Bakhtiyarpur (Saharsa district) respectively. Significantly higher estimate for Simri Bakhtiyarpur (p<0.05) than HMIS-based estimate was in concordance to earlier studies conducted in India. Singh VP *et al*., reported underreporting of kala-zar cases by a factor of 4.17 [18]. In a study conducted in East Champaran district of Bihar, Das P *et al*. estimated annual incidence of 35.6, 16.8 and 21.9 cases per 10,000 population for a high endemic PHC, medium endemic PHC, and overall at district level respectively, which was much higher than officially reported incidence rate [19].

The possible overall estimates for higher endemic PHC’s of Bihar State was 10.4 per 10,000 population, which was significantly higher (p<0.001) with that of 2.3 per 10,000 for Bihar state (As per the surveillance data of the country; NVBDCP). There are no estimates available for higher endemic PHCs, neither at national level nor at the state level. None of the research study provides such estimates for Bihar state. Both the PHCs under the present study were well above the elimination level as per the estimated annual incidence computed by both house-to-house and snowballing approach. Although the possible estimates found under this study have low precision, but it may provide a lead to kala-zar disease burden at least for higher endemic sub-districts/ PHCs of Bihar. The probable factors for this low precision may be sample size considered, selection of study blocks, and underreporting of cases. High precision estimate can be achieved through large sample size.

The present study aimed to assess efficacy of snowballing approach as an alternative to the highly exhaustive and costly house-to-house survey approach. As compared to HHA, snowballing approach was able to capture about 50% of kala-azar cases. However, SBA captured deaths more efficiently (76%) as compared to capturing of cases. It is noteworthy to discuss here that in spite of uniform educational criteria for recruitment of snowballing team members, training and logistic support, out of 10 teams (numbered as Team-1 to Team-10), Team-3 (Lalganj PHC) and Team-9 (Simri Bakhtiyarpur PHC) had slightly higher performance than rest of the teams. This inter-team performance variation attracts more careful recruitment of personnel for better performance of SBA.

House-to-house survey, the “Gold-standard” tool, captured most of the cases missed by SBA with some percentage of omission (about 13%). It may be related to wrong information from fellow villagers, non-availability of respondents, hurriedness of field investigators in conducting the survey, non-coverage of isolated clusters of villages, and slackness in monitoring and supervision etc. The wrong information from fellow villagers may be linked to lack of appropriate knowledge about kala-azar symptoms amongst general population. Under the present study, level of knowledge, attitude and practice (KAP) at the community level towards kala-azar was not assessed. However, proper information, education and communication (IEC) activity at community level may enhance the sensitivity of both HHA and SBA.

Apart from effective strategies for diagnosis, treatment and control, community participation in programme is a key for success of national kala-azar elimination programme. The key informants grouped under “defined (accredited)” category were supposed to be potentially competent informants for snowballing approach. But statistically significant difference (p<0.001) was observed between “other” categories of key informants and “defined (accredited)” categories of key informants in identification of kala-azar cases and deaths. It suggests more efforts to be undertaken to strengthen the defined categories of personnel. Poor performance of ASHAs in identifying kala-azar cases differed from the earlier conducted study by Das et al., 2014 in India [20]. However, ASHAs were found more effective (7.02%) in identifying deaths. Nowadays, there is provision of incentives for ASHA for detection of new VL case after ensuring complete treatment. However, the study finding suggests that still ASHAs need to be motivated towards kala-azar programme. Some of the reasons may be: lack of training, re-orientation, level of understanding about kala-azar disease, non-payment of incentives in time for their activities, etc. Possible identification of kala-azar cases (only 1.2%) by village leaders/Sarpanch indicates their less involvement in kala-azar programme, weak and ineffective awareness strategies at the community level, weak social mobilization, etc. Their position and service may be utilized in more effective way by the national policy and program managers for kala-azar control at the village/Panchayat level. Further, in spite of defined categories of key informant other undefined categories of key informant need to be visualized and streamlined to strengthen the kala-azar elimination programme and thus to achieve the elimination goal.

The socio-demographic characteristics of kala-azar cases identified during the survey revealed that significantly higher proportion (268; 48%) of kala-azar cases were from 0–14 years of age as compared to rest age-groups viz. 15–29, 30–44 & 45–59 years (p<0.001). About half of the VL cases were from growing age group and majority (333; 59%) was males. Kala-azar patients coming from very low socio-economic categories (viz. labourers (unskilled), rickshaw pullers, Agricultural labourer, etc.) were significantly higher (78%) as compared to middle and higher socio-economic (as combined) categories of patients (p<0.001). The socio-demographic characteristics found under this study was almost identical at par with other studies conducted in India [21–23].

The concordance between Health Management Information System (HMIS) and house-to-house data revealed that about 75% of cases detected by house-to-house approach were completely and accurately matched with HMIS data and rest 25% cases were not reported to HMIS. This finding suggests that still there is underreporting of kala-azar cases. Hence, there is a need to further improve the kala-azar surveillance system to report the actual disease burden.

In terms of cost analysis, the ratio of man-days for snowballing and house-to-house approach per PHC was 1:6 and snowballing cost was about just half when compared to house-to-house approach. Though snowballing approach was not found sensitive enough but it was found as a cost-effective alternative to HHA.

Keeping in view assessment of kala-azar elimination programme in India, snowballing approach may be used as an alternative approach after standardization based on the strengths and weaknesses of this study finding. As snowballing approach correctly recorded about 77% kala-azar deaths, it may be considered more appropriate especially in rural areas for capturing deaths due to kala-azar and could be used as an effective methodology for enumerating annual morbidity and mortality of kala-azar.

### Conclusions

In our study, though snowballing sampling approach was not found sensitive enough but it was rapid and cost effective as compared to house-to-house survey approach. The possible reasons for failure of snowballing approach in the present study might be lack of adequate training, competency, motivation to field investigators, access to the isolated clusters, high level supervision and monitoring, etc.

As annual incidence is the key indicator for monitoring progress of the national kala-azar elimination programme, kala-azar surveillance system needs to be strengthened. Presently, kala-azar control program moves to an elimination trajectory, hence it needs to be evaluated in the country by the end of extended target year 2017. Such assessment would be resource extensive and require huge logistics and finance. Under the above situation, snowballing approach may be used in future as a cost-effective surveillance approach after standardization of specific procedure, training and logistics.

### Limitation of the study

This study was conducted in large sample population (thorough coverage of two PHC’s areas) as compared to earlier studies conducted in India. The estimated incidence by this study may lack precision due to requirement of sample size. Possible reasons for missing of cases include–absence family members at the time of visit by team, concealing of fact by respondents due to fear/stigma, households left by team, etc. Discordance between house-to-house and snowballing approach may be due to different response by the respondents to different teams.

## Supporting Information

S1 File Snowball sampling procedure for identification of potentially suspected case of kala-azar in a village. (DOC)

## References

[1] Stauch A, Sarkar RR, Picado A, Ostyn B, Sunder S, Rijal S, et al Visceral leishmaniasis in the Indian subcontinent: modelling epidemiology and control. PLoS Negl Trop Dis. 2011; 5(11): e1405 doi: 10.1371/journal.pntd.0001405 22140589 10.1371/journal.pntd.0001405 PMC3226461

[2] Bern C, Chowdhury R. The epidemiology of visceral leishmaniasis in Bangladesh: prospects for improved control. Indian Journal of Medical Research. 2006; 123(3): 275–288. 16778310

[3] Joshi A, Narain JP, Prasittisuk C, Bhatia R, Hashim G, Jorge A, et al Can visceral leishmaniasis be eliminated from Asia? J. Vector Borne Dis. 2008; 45(2): 105–111. 18592839

[4] World Health Organization: Regional Strategic Framework for Elimination of Kala-azar from the South-East Asia Region (2011–2015), New Delhi: Regional Office for South-East Asia, 2014.

[5] A consultation meeting on indicators for Kala-azar elimination organized at SEARO office, New-Delhi in June 09.

[6] Brain storming session meeting at National Vector Borne Diseases Control Programme office, New-Delhi on 19th Jan. 2011.

[7] National Vector Borne Disease Control Programme, Directorate General of Health Services, Ministry of Health & Family Welfare, Kala-azar disease burden in India: www.//http://nvbdcp.gov.in.

[8] Singh SP, Reddy DCS, Rai M, Sunder S. Serious underreporting of visceral Leishmaniasis through passive case reporting in Bihar, India. Tropical Medicine and International Health. 2006; June,11: 899–905. 10.1111/j.1365-3156.2006.01647.x 16772012

[9] McNamara RP. Male Prostitution in New York City,Westport: Praeger.The Times Square Hustler. 1994.

[10] Avico U, Kaplan, C.Korczak, d. Van Meter, K. Cocaine. Epidemiology in three European Community cities: a pilot study using snowball sampling methodology, Brussels. European Communities Health Directorate. 1988.

[11] Griffiths P, Gossop M, Powis B, Strang J. Reaching hidden populations of drug users by privileged access interviewers: methodological and practical issues. Addiction. 1993; 88: 1617–26. 8130701 10.1111/j.1360-0443.1993.tb02036.x

[12] Kaplan CD, Korf D, Sterk C. Temporal and social contexts of heroin-using populations: an illustration of the snowball sampling technique. J Nerv Ment Dis. 1987; 175: 566–74. 3655783 10.1097/00005053-198709000-00009

[13] Sudman S, Freeman H. The use of network sampling for locating the seriously ill. Med Care. 1988; 26: 992–9. 3172869 10.1097/00005650-198810000-00007

[14] Snijders T. Estimation on the basis of snowball samples: how to weight? Bull Methodol Sociol. 1992; 36: 59–70.

[15] Singh Padam, Pandey Arvind and Aggarwal Abha. House-to-house survey vs. snowball technique for capturing maternal deaths in India: A search for a cost-effective method Indian J Med Res. 2007; 125:550–556. 17598941

[16] Institute for Research in Medical Statistics. Report of Estimates of Maternal Mortality in Five States of India: A Pilot Study. 2002; http://www.icmr.nic.in.

[17] Office of the Registrar General & Census Commissioner, India, Ministry of Home Affairs, Government of India, New-Delhi. Website: https://data.gov.in.

[18] Singh VP, Ranjan A, Topno RK, Verma RB, Siddiqui NA, Rabidas VN, et al Short Report: Estimation of underreporting of visceral leishmaniasis cases in Bihar, India. American Journal of Tropical Medicine and Hygiene. 2010; 82(1): 9–11. 20064987 10.4269/ajtmh.2010.09-0235 PMC2803501

[19] Das P, Samuels S, Desjeux P, Atul Mittal, Roshan Topno, Niyamat Ali Siddiqui, et al Annual incidence of visceral leishmaniasis in an endemic area of Bihar, India. Tropical Medicine and International Health. 2010;15 (2): 4–11. 20487422 10.1111/j.1365-3156.2010.02517.x

[20] Ravi Das Vidya Nand, Pandey Ravindra Nath, Pandey Krishna, Singh Varsha, Kumar Vijay, Matlashewski Greg, et al Impact of ASHA Training on Active Case Detection of Visceral Leishmaniasis in Bihar, India. PLOS Neglected Tropical Diseases. 2014; 8 (5): e2774 doi: 10.1371/journal.pntd.0002774 24853122 10.1371/journal.pntd.0002774 PMC4031043

[21] Sheets D, Mubayi A, Kojouharov HV. Impact of socio-economic conditions on the incidence of visceral leishmaniasis in Bihar, India. Int. J Environ Health Research. 2010; 20(6):415–30. 10.1080/09603123.2010.491853 21161803

[22] Thakur C.P. Socio-economics of visceral leishmaniasis in Bihar (India). Trans RSTMH. 2000; 94 (2):156–157. 10.1016/s0035-9203(00)90255-4 10897353

[23] Sarnoff Rhonda, Desai Jaikishan, Desjeux Philippe, Mittal Atul, Topno Roshan, Siddiqui Niyamat Ali, et al The economic impact of visceral leishmaniasis on rural households in one endemic district of Bihar, India. Tropical Medicine and International Health. 2010;15(2): 42–49, 20487423 10.1111/j.1365-3156.2010.02516.x

