# Supplementary material for: Snowball Vs. House-to-House Technique for Measuring Annual Incidence of Kala-azar in the Higher Endemic Blocks of Bihar, India: A Comparison
Source: PLoS Negl Trop Dis. 2016 Sep 28;10(9):e0004970. doi: 10.1371/journal.pntd.0004970 (PMC5040448; doi:10.1371/journal.pntd.0004970)
Supplement: S1 File — (DOC) [file pntd.0004970.s001.doc]

**Snowball sampling procedure for identification of potentially suspected case of kala-azar in a village**

- Walked to roughly the centre of the village.
- Gathered few people (adult men and women) and introduced our self as from the health department and asked for basic details of the village, sketched a rough map with their help, and marked relevant locations.
- Inquired about:
- How large is the village? How many houses? How many people?
- How many different neighbourhoods? How many different caste-groups? Where do they stay? How many houses in each caste-group? How far from here?
- Is there any outlying *tolas*? If yes, how many, how large, does it belong to this village?
- How many Aganwadi centres (AWC) in the village? In which part of the village are they located? Where can they be found now?
- How many ASHA in the village? Where do they live? Where can they be found now?
- Any ANM/sub-centre in the village? Where does the ANM live? Where she is available now?
- Who is the Sarpanch/ leader of the village? Where does he/she live?
- If these informants appeared to be knowledgeable about the village, asked further disease-specific questions as below, otherwise contacted other informants nearby
- When people of the village fall ill, say, with fever, where do they go for treatment? Is there anyone in the village to whom people consult? Is there any local healer? Where he/ she is available in the village?
- If someone has Kala-azar, where would he/she be taken?
- Do you know anyone who suffered from Kala-azar in the village in the last two years or anyone who has/ had prolonged fever? Any person who was sick (fever) for a long time and could not diagnosed? Someone who had recovered from illness?
- Is there any death in the village due to illness in the last two years?
- Based on this information, visited each neighbourhood/ caste-group/ tola of the village.
- Gathered a few villagers (both sexes), who were not the key informants, and asked them about disease-specific questions as above.
- Reviewed the map and information table and see if any key informant has been marked in this *tola*. Ask for their homes and visit each of them. If no one has been marked, ask – Is there any ASHA or AWW living in your *tola*?
- Updated sketched map and information table for identified potentially suspected (PS) cases and deaths. Visited the PS home for interview and identification of another PS case.
